# Patient engagement in Canada: a scoping review of the ‘how’ and ‘what’ of patient engagement in health research

**DOI:** 10.1186/s12961-018-0282-4

**Published:** 2018-02-07

**Authors:** Elizabeth Manafo, Lisa Petermann, Ping Mason-Lai, Virgnia Vandall-Walker

**Affiliations:** 1 Patient Engagement Platform, Alberta SPOR SUPPORT Unit, Faculty of Health Disciplines, Athabasca University, 1 University Drive, Athabasca, Alberta T9S 3A3 Canada; 2 Patient Engagement Platform, Alberta SPOR SUPPORT Unit, Faculty of Health Disciplines, Athabasca University, 1 University Drive, Athabasca, Alberta T9S 3A Canada

## Abstract

**Background:**

Over the last 10 years, patient engagement in health research has emerged as the next evolution in healthcare research. However, limited evidence about the clear role and scope of patient engagement in health research and a lack of evidence about its impact have influenced the uptake, implementation and ongoing evolution of patient engagement. The present study aims to conduct a scoping review to identify methods for and outcomes of patient engagement in health research.

**Methods:**

An adaptation of the scoping review methodology originally described by Arksey and O’Malley and updated by Levac, Colquhoun and O’Brien was applied. Sources from a formal database search and relevant documents from a grey literature search were compiled into data extraction tables. Articles were synthesised into key themes according to the (1) methods and (2) outcomes of patient engagement in health research.

**Results:**

The total yield for the scoping review was 55 records from across Canada, the United Kingdom and the United States. While evidence about the methods used to engage patients in health research is increasing, stronger evidence of specific patient and healthcare system outcomes is required. This necessitates further mobilisation of research that explores outcomes and that validates specific tools to evaluate engagement. Additionally, theoretical frameworks that can better inform and sustain patient engagement across the lifecycle of health research are lacking.

**Conclusion:**

Further increasing the volume and reach of evidence about patient engagement in health research will support the paradigmatic shift needed to normalise the patient’s role in research beyond ‘subject’ or ‘participant’, so as to ultimately improve patient health outcomes and better address healthcare reform in Canada.

## Key Messages

- Engaging patients across the research continuum provides positive opportunities for the patient, researcher and healthcare system to improve patient and healthcare outcomes together.
- Existing efforts to engage patients are often limited to preliminary activities that are not sustained across the research activity spectrum. This is largely related to barriers in the process, including limited availability, awareness and understanding of guiding frameworks and validated methods, as well as resource constraints such as time and money, for adequate planning.
- Evaluation frameworks and sufficient evaluation data to measure near, intermediate and long-term outcomes of engaging patients across health research activities are needed. Successful and sustained adoption of meaningful engagement is hinged on reliable outcomes.
- Efforts are already underway to better engage with patients in health research in the United Kingdom, United States and Canada. The time to secure this paradigmatic shift is now, while momentum has been built and funding has been designated to further sustain such opportunities.

## Background

Advances in technology have increased access to information, resulting in patients being more informed than ever about their conditions and care options. Including patients’ active voices is becoming the espoused healthcare ideology, which has crucial implications for patient experiences, health outcomes, and research and healthcare funding [1]. Patient engagement is considered a precursor to quality, patient-centred care, which ultimately drives quality improvement [2–6]. The outcome of patient-centred care provides a ‘triple aim’ impact on (1) a patient’s experience of care, (2) patient outcomes at the individual and population level, and (3) per capita healthcare costs [4].

Increased interest in patient engagement comes at a time when healthcare spending is steadily increasing in Canada – in 2016, it is estimated that health expenditures reached $228 billion, representing 11% of Canada’s Gross Domestic Product [7, 8]. As such, ensuring sustainability and growth of health services remains a top priority in Canada [8]. It has also been suggested that a loss of confidence in the Canadian healthcare system requires increased public engagement to invigorate vitality into healthcare reform [9].

A recent component of this ongoing commitment to patient engagement is the increased interest and investments specifically for patient engagement in health research. The premise for engaging with patients beyond the level of research subjects or participants reflects a growing desire for more ethical, democratic and moral healthcare practices as well as for a more informed and accountable research agenda [10–14]. Moving away from health discipline paternalism [13] is more than simply a means to advance a novel research paradigm [15]. Instead, this paradigmatic shift towards patient engagement in health research opens opportunities for greater impact on the ‘triple aim’ of patient-centred care. This shift demands that researchers, clinicians and healthcare administrators view patients as active and respected partners in the research process. Indeed, patients are the ultimate recipients of health research findings, and thereby the most important stakeholders [16, 17]. In Canada, the Canadian Institute for Health Research’s Strategy for Patient Outcome Research (SPOR) defines patient engagement in health research as “*occur*[ing] *when patients meaningfully and actively collaborate in the governance, priority setting, and conduct of research, as well as in summarizing, distributing, sharing, and applying its resulting knowledge*” [18]. Globally, however, the language used to define patient engagement differs. For example, in the United Kingdom, ‘patient and public involvement’ is the preferred language because it denotes how members of the public are “*actively involved in research projects*” [19]. Despite these differences, the literature generally supports that ‘patient’ should not be solely defined as an individual receiving care. Instead, the term refers to any individual or group with lived experience of a health or health systems issue, including family members, caregivers and the organisations that are involved with the population of interest [19, 20].

In the last 10 years, patient engagement in health research has emerged as the next evolution in healthcare delivery; an opportunity to involve patients in decision-making related to health research while improving health outcomes [10, 21, 22]. There are several arguments for advancing patient engagement in health research that are underpinned by moral, consequential and ethical value systems [13, 15, 23, 24]. Furthermore, incorporating the patient perspective of ‘nothing about me, without me’ has helped address the increased pressure for greater accountability for public spending and a stronger focus on outcome measurement [21, 25, 26]. Despite these overarching values, there is limited consensus on how to engage patients throughout the research process, resulting from the limited understanding of the meaning, conceptualisation and activities of engagement, thereby contributing to a reluctant uptake of this type of engagement [15, 27, 28].

Furthermore, limited evidence of the clear role and scope of patient engagement in research is coupled with a lack of evidence about its impact [15, 29–32], resulting in a ‘catch-22’ situation. This has rate-limiting implications; that is, the knowledge-to-action gap [29] between the ‘what’ and ‘how’ of patient engagement in research, as well as the interrelationship between them, may potentially limit its implementation, ongoing evolution and uptake by health research disciplines [33–35]. This could be in part due to the several actual or perceived barriers to engaging patients in research, including an increase in resources required [30, 35–39], the possibility of patient voices inadvertently shifting the research agenda away from original purpose [15, 40, 41], a lack of supportive infrastructure and culture [21, 29], a lack of clarity in taxonomy of patient engagement in health research [14, 42, 43], tension between physicians and patients [44], and tokenism [15, 30].

There is therefore a critical opportunity for researchers to focus on validating frameworks and methods to support meaningful patient engagement in health research. It is important to build on existing evidence of what works in achieving and sustaining productive patient engagement [17], and what does not [1, 45], in order to evaluate whether meaningful patient engagement in the research enterprise impacts enhanced patient-centred care, service delivery, health outcomes, and healthcare costs.

As such, the Patient Engagement Platform, one of seven platforms of the Alberta SPOR SUPPORT Unit, commissioned a review of the literature with the objectives of identifying (1) the origins of patient engagement and how this impacted the conceptualisation of patient engagement in health research, (2) the methods used to engage patients in health research, and (3) the outcomes of patient engagement in health research. For the purpose of this paper specifically, the authors will focus the findings on the methods used and outcomes of patient engagement in health research. The conceptualisation of patient engagement in health research has been described in detail elsewhere [46].

## Methods

An adaptation of the scoping review methodology originally described by Arksey and O’Malley [47] and updated by Levac, Colquhoun and O’Brien [48] was adopted to review the literature on patient engagement in health research. This approach aims to identify and map relevant literature in a particular field that (1) is in its infancy and/or (2) is too heterogeneous for the conduct of a systematic review. It was determined that this methodology was the most suitable to identify, extract and summarise literature about patient engagement in health research because it appropriately aligns with both criteria.

### Search matrix

Based on consultation with the Project Advisory Committee, composed of the members of the Patient Engagement Platform in Alberta, the following research questions were identified: What are the origins of patient engagement in healthcare and how did it impact the conceptualisation of patient engagement in health research? What are the methods of patient engagement in health research? What are the impacts of patient engagement in research, if any, in promoting the health of people and improvements to the healthcare system?

The following assumptions were made to further clarify the definitions of commonly used terms when formulating the research questions: (1) ‘patient’ is used as an overarching term to include individuals with personal experience of a health or health systems issue, and informal caregivers, including family and friends; (2) ‘patient engagement’ refers to meaningful and active collaboration in methods of engagement, which include governance, priority setting, conducting research, knowledge translation and evaluation; and (3) impacts on patient health and the health system, as categorised using the Patient-Centered Outcome Research Institute’s levels of impact and the Canadian Institute for Health Research’s SPOR Patient Engagement Outcomes described in Table 1.

**Table 1:** Impacts on patient health and healthcare system matrix

PCORI Level of Impact	SPOR PE Outcomes
Long-term (system)	● A contribution to improving the cost-effectiveness of the healthcare system
Intermediate (organisation)	● The right treatment at the right time ● Improved access to the healthcare system ● Active and informed patient partnerships in healthcare
Near-term (individual)	● Improved health ● Quality of life that is tied to patient-oriented outcomes ● Active and informed patient partnerships in healthcare

*PCORI* Patient-Centered Outcome Research Institute, *PE* patient engagement, *SPOR* Strategy for Patient Outcome Research

SPOR’s principle objective is to enhance the volume and quality of patient-oriented research, thereby bringing innovative approaches to the point of care while ensuring greater quality, accountability and accessibly of care for all Canadians [49]. The authors acknowledge that there are other relevant patient engagement frameworks that have a more detailed focus on outcomes – in the United States (PCOR) and in the United Kingdom (INVOLVE). The SPOR patient engagement framework and patient outcomes were specifically selected for this review in order to contextualise the results of the emergent focus on patient engagement in health research in Canada. Given the guiding principles embedded in SPOR’s framing of patient engagement in health research (i.e. moving from level of ‘subject’ to ‘partner’), the findings of this review are to be considered against what would ultimately matter to the patient themselves.

Specific and appropriate electronic databases were identified to explore the research questions with the assistance of Ryerson University Library Services, Toronto, Canada. The four selected databases were HealthStar (OVID), CINAHL, Scholar’s Portal and Proquest. Next, keywords were identified based on a preliminary scan of the literature, which established a basic understanding of the typical lexicon used in this research area. The search strategy used for each database was comprised of the search terms ‘patient’ OR ‘public engagement’, ‘participation’ (MeSH), ‘involvement’, ‘activation’ OR ‘patient engagement in research’ AND ‘health’ AND ‘health care delivery’. Search terms were adapted as needed to best meet the requirements of each database. Additional filters (e.g. geography, year of publication, language) were applied when available in the databases to further refine that the search and yield were appropriate to the inclusion and exclusion criteria described. The following selection criteria were established based on the purpose and scope of the search. To be included in the review, articles had to be (1) peer-reviewed journal articles, research reports or guideline documents; (2) published in English or French; (3) published within the timeframe of 2006 to 2017 (11 years); and (4) studies conducted in Canada, United States, Europe, United Kingdom, Australia and New Zealand.

Of those articles meeting the inclusion criteria, those that were focused on (1) client/community-based engagement beyond primary health research and (2) patient education/patient care or patient engagement in client care (e.g. Patient Portals, eMRs) to support decision-making/improvements in quality of care/uptake of care/intervention, were excluded.

### Search strategy

A formal and informal search was included as part of the scoping review methodology. The formal search using the inclusion and exclusion criteria, involved scanning the articles using a four-step process:

1. Article identification: records were screened by title and those considered irrelevant were excluded (records that could not be identified through title screen only moved to the second stage);
2. Article screening: records were screened using title and abstract and excluded if they were not relevant (incorrectly moved from step 1), or were duplicates (determined through manual search);
3. Article assessment: records were assessed for selection and quality appraisal criteria and excluded if they did not meet the criteria. To further expand the yield of the formal search, a subsequent manual search was conducted to help identify any additional articles relevant to the research questions. For those articles meeting the selection criteria, the reference list of key articles were reviewed and key articles were entered into the ScienceDirect formal database citation matcher to see if additional relevant articles were identified;
4. Articles selected: articles that passed through each subsequent step were organised based on the three identified research questions. Articles with a primary focus that addressed the research question were organised appropriately, acknowledging the overlap between each focus area.

To deepen the reach of the proposed literature search, an informal grey literature search was conducted (i.e. un-catalogued research) using a defined process. First, grey literature repositories relevant to health and public health disciplines [36] were reviewed and four were selected as appropriate for the specific literature search across North America and the United Kingdom; these were desLibris, Health System Evidence (Canadian), Open Grey (United Kingdom), and the Agency for Health Care Research and Quality (United States).

In addition, Canadian, United States and United Kingdom health research and policy organisations were systematically explored with more general, yet limited in number, search terms from the formal database search to increase the likelihood of finding a relevant yield (i.e. ‘patient engagement’ or ‘patient engagement research’). Searches were limited to up to 100 hits per website query.

### Data extraction

Research articles from the formal database search and relevant documents from the informal, grey literature search were compiled into data extraction tables. Quality assessment criteria were used to assess academic literature [37, 38] and grey literature [39]. For the selected articles, data extracted included the resource citation, type of resource, key publishing authors, geographic location, study setting, population of focus, study methodology, context/impetus for research, preferred patient engagement language/terminology and key research outcomes. When available, characteristics of the patient engagement methods, limitations and/or barriers to implementing a patient engagement framework and impact of patient engagement in research were also included.

## Results

**Fig. 1:**
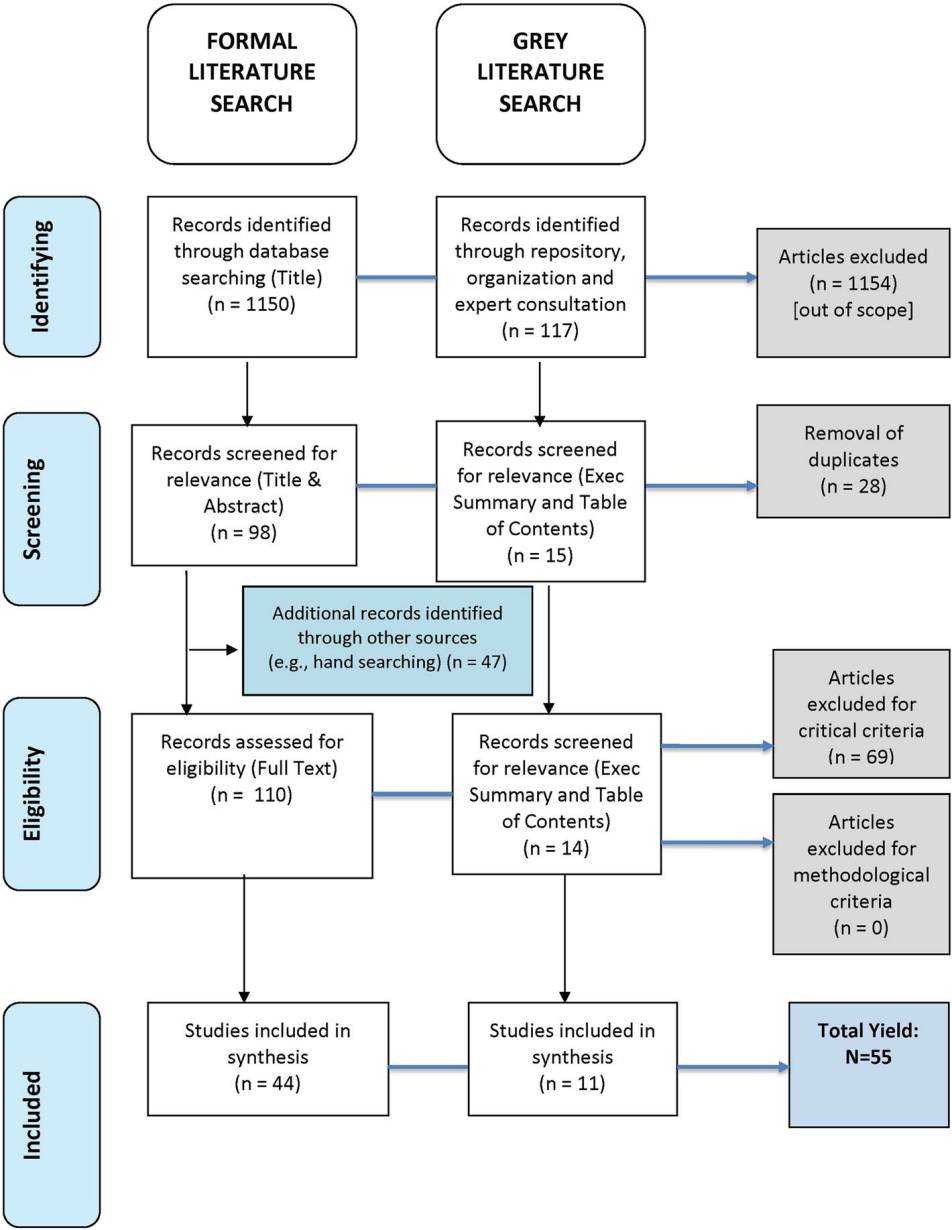
Article selection flow diagram

The complete article yield across the three research questions was 77. Excluding the articles that solely focused on the conceptualisation of patient engagement, the total yield for this scoping review was 55 records (n = 44, formal review; n = 11, informal review/grey literature). In Fig. 1, the flow of articles identified, screened, selected and reviewed are described. The terminology for patient engagement varied considerably, with a wide range of terms used. The most common terminology used included ‘patient and public involvement’ (n = 14) and ‘patient engagement’ (n = 12), whereas ‘patient involvement’ (n = 4), ‘stakeholder engagement’ (n = 4), ‘patient participation’ (n = 2) and ‘patient oriented research’ (n = 2) were less commonly applied. Other terminology like ‘patient activation’, ‘patient and clinical engagement’, ‘citizen engagement’, ‘patient or consumer involvement’ and ‘patient research’ were used once. Several authors [13, 27, 43, 50, 51] confirmed a need to have clear, consistent terminology to denote patient engagement, which can be used and applied across various contexts to inform a clear conceptualisation and understanding of patient engagement across the research process.

An overview of the selected record (academic and grey literature) characteristics is described in Table 2.

**Table 2:** Overview of selected record characteristics

Characteristic	Output
Record type	● Journal article (n = 44) ● Research report (n = 1) ● Guidelines document (n = 8) ● PowerPoint Webinar presentation (n = 2)
Methodology (formal literature only)	● Case study/series (n = 7) ● Experimental (n = 1) ● Quasi-Experimental (n = 2) ● Non-experimental (n = 15) ● Qualitative interviews (n = 7) ● Literature review (n = 11) ● Commentary/Editorial (n = 1)
Year	● Last 5 years (2013–2017) (n = 33) ● 5–10 years (2006–2012) (n = 19) ● No date (n = 3)
Geography	● Canada (n = 19) ● United Kingdom (n = 17) ● United States (n = 13) ● Europe (n = 0) ● Australia/New Zealand (n = 0) ● Netherlands (n = 6)

The results were further examined by the methods (the ‘how’) and reported outcomes (the ‘so what’) of patient engagement in health research.

### The ‘how’: methods for patient engagement in health research

A description of the patient engagement research lifecycle, levels of engagement in health research and stakeholders needed to promote patient engagement in health research are described elsewhere [15, 40, 52].

A recent systematic review [36] of 142 articles wherein patients were engaged across research activities, did not result in the authors recommending any one particular method of choice to support effective patient engagement in research. Given the infancy of this health research practice, tracking published experiences of engaging patients may help in comparing trends in patient engagement in health research that are most feasible until a stronger evidence-base is developed [3, 41]. Recent studies have begun initiating the dialogue of ‘how’ patients can be involved throughout the research lifecycle [21, 53, 54]. Patient engagement initiatives (or programmes) in the United States and United Kingdom have also included development of comprehensive guides for researchers. Based on the literature findings, we focused on those approaches considered successful in the literature to supporting patient engagement in health research (Box 1). These approaches, considered for inclusion early in the research lifecycle (e.g. preparation and planning), were (or could be) also facilitated and fostered throughout (e.g. study design, analysis and dissemination) in order to capture and optimise the patient perspectives across all phases of health research.

**Box 1** Successful engagement approaches for patient engagement in health research [15, 30]

**Table:** 

1. Engage patients as early as possible and continue engagement throughout
2. Clearly define patient engagement plan; be clear on roles, duties and expectations between patients and researchers
3. Provide orientation and education about research and patient engagement
4. Provide ongoing support, encouragement and recognition for patient contributions
5. Facilitate mutual respect and valuing of patients’ expertise based on knowledge gained through experiences
6. Ensure a trusting and positive environment by providing structural support
7. Include a plan for evaluation of engagement

In addition to the successful engagement approaches, the literature also provided shared characteristics of successful patient engagement opportunities in health research (Box 2).

**Box 2** Shared characteristics of successful patient engagement in health research

**Table:** 

• Clear purpose, role and structure for engaging patients [26, 55]
• Initiate and maintain partnerships between researchers and stakeholders [24, 42, 44, 55]
• Take the time required to foster relationship-building as the most critical component in establishing trust [55–57]
• Clear leadership from principal investigator and/or wider culture of involvement [56]
• Promote the need for facilitation of cross-communication among all groups [37, 44, 55]
• Capture and optimise patient perspectives across all phases of research [44, 55, 58]
• Ensure meaningful patient influence on research by validating the need for respect and support for patients [37, 44, 59, 60]
• Ensure adequate training for researchers and patients [44, 59]
• Share and promote research learnings, including evaluation efforts [37, 44, 55, 58–60]

Still, there is infrequent engagement of participants, particularly across the research lifecycle. In a recent review article [61], authors identified nearly 200 studies involving Patient Reported Outcome Measures for chronic disease and quality of life impact. Yet, only 30% included patients in the research process at all, and fewer than 10% of these patients participated across the entire research activity spectrum. further limiting the potential impact of engagement. The uptake of patient engagement in research however, is slow; only a small proportion of the general public have participated personally in research projects or have known someone who has participated in patient engagement in health research [15, 33, 53, 62].

### The ‘so what’: outcomes of patient engagement in health research

The literature related to the outcomes of patient engagement in health research is less clear. There is a growing consensus that integrating the patient voice into the research lifecycle increases the legitimacy and rationality of decision-making, while improving the overall quality and applicability of outcomes [21, 63], including client-centred care [8]. This is documented by several impacts of patient engagement in research based on reports of positive outcomes for patients [10, 12, 16, 21, 27, 30, 44, 55] and impact on the research itself, including its design, delivery and application (i.e. improved research quality, increased translation and mobilisation of knowledge, expanded applicability of research) [10, 12, 15, 40, 41, 55, 56, 64]. These first person reports are summarised in Table 3.

**Table 3:** First person reports of patient and researcher outcomes when engaging patients in health research

Outcomes for patients	Outcomes for researchers
• Patient developed own voice and agenda; patient was more prepared for broader collaboration with other stakeholder groups [21, 40] • Patients felt empowered, valued, and gained confidence and life skills [10, 28, 40] • Researcher and patient developed improved trust [55] • Improvement in information on all aspects of disease and treatment, involving patients in decision-making, organisation of care and the burden of neuropathy; setting [33, 55] • Improvement in quality of care in context of research priority setting [33]	• Increased enrolment in studies and decreased attrition; improved data collection tools; improved dissemination of study findings and mobilisation of findings [15, 36, 50, 70] • Greater understanding and insight into research area; rapport with community built [40, 55] • Better alignment of research objectives through priority-setting activities [10, 33, 38, 71, 72] • Improved research effectiveness [15, 55] • Improved opportunity to appraise and evaluate engagement opportunities in research [55]

While there is evidence in the literature of the merits of patient engagement for improving the effectiveness of the research process, the content and quality of the articles providing this evidence was highly variable. Much of the purported benefits of patient engagement for impacting broader health outcomes and healthcare reform do not rely on experimental studies that have been assessed or formally evaluated by any formal measures of evaluation [11]. Several studies had limited or poorly described methods of assessment and evaluation [15, 50, 65]. Further, the studies that did evaluate impact were limited to qualitative analyses of small samples, which limits the generalisability of the findings [10].

In reference to SPOR’s identified desired outcomes, the literature most often revealed near or intermediate outcomes related to ‘being an active and informed partner in healthcare’, yet otherwise revealed limited impact on near, intermediate or long-term outcomes [10, 32, 41, 59, 65, 66]. Often, the emphasis is on impacting the effectiveness and efficiency of the research process itself, with little to no translation across actual implementation and evaluation of research findings. However, more recent studies in the United Kingdom are aiming to strengthen evaluative measures to ensure meaningful impact on patient outcomes, the scope of which differ from those of SPOR [55].

## Discussion

Engaging patients across the research lifecycle opens up positive opportunities to ultimately improve patient and healthcare outcomes [26]. This has translated into the development of strong programmes for patient engagement in health research in the United Kingdom, United States and Canada. A cornerstone of the rhetoric of patient engagement in health research is providing patients with opportunities for building capacity for active participation that goes beyond their personal health decision-making and into framing healthcare for other patients and populations [15, 46, 60].

While current momentum is high, several challenges exist in normalising patient engagement within the context of every day research. Over 10 years ago, Hewlett et al. [59] identified the challenges and benefits of engaging patients and the need for a clear, practical and feasible framework to guide the process. The absence of a validated framework to guide patient engagement in health research specifically is limiting adoption across the research lifecycle. Existing efforts are focussed on ‘lessons learned’ [21, 40], recommendations [30] and checklists to support patient and public involvement [53]. This may result in outcomes that are inadequate or ineffective considering the breadth of outcomes at the individual, organisational and system levels. Building a robust patient engagement enterprise requires a firmer and more widespread understanding by both researchers and patients of the ‘how’ to effectively and efficiently include patients in a meaningful and feasible way [30, 36].

Reporting on the identified levels of outcomes is needed to demonstrate the ‘return on investment’ of engagement in research [17, 63, 67]. Innovative indicators that accurately capture the costs and benefit of patient engagement are needed to best attract researchers [26, 54, 62], patients, clinicians and healthcare administrators in seeing the value of this practice, including its value in the development of patient-informed and patient-reported outcomes [54, 62]. As demonstrated [10, 15, 41], existing efforts to engage the patient are often limited to preliminary activities that are not sustained across the research activity lifecycle [10, 15, 26, 36]. Given the weak evidence base, engagement in research is at risk of being driven more by a promise that research and health outcomes will improve [10] and, as such, the initiative will have many detractors. For a culture shift to occur, a solid theoretical and practical framework needs to be established and supported by sufficient data to legitimise and sustain the initial investment and dedication of researchers and funding institutions. This requires a direct focus on addressing the fragmentation in patient engagement in health research by focusing on its poor conceptualisation and understanding, inconsistently applied frameworks and limited emphasis on evaluating the impact of involvement [68]. To further portray this enterprise of patient engagement in health research, this fledgling initiative can ill afford a lack of robust evidence that underlies the impetus supporting patient engagement in research [63, 68].

## Limitations

The literature included in this review was largely conducted in the United Kingdom and the United States, where impact on the healthcare delivery and research process may differ from that in Canada (e.g. Medicare values and principles of the Canada Health Act). Such considerations will influence the interpretation of the findings.

Second, the search was limited to specific databases between 2006 and 2017 and grey literature sources that were informed by a specific search matrix, which may have limited the findings reported in this review. An attempt to minimise this limitation was addressed by completing a hand search using record reference lists and the single citation matcher function to search for other related and relevant articles.

Similarly, given the breadth of patient engagement literature, the intent of this review was to refine literature findings to support exploration of the identified research questions. The evolution of patient engagement over the last 11 years and the inconsistencies in terminology may have unintentionally resulted in articles being excluded. For example, ‘consumer’ was not included in the search terms as an alternative to ‘public’ or ‘patient’, which may have limited the findings. Furthermore, if global interventions relating to patient engagement were explored in more depth, key terms would also include specific and established patient engagement processes, like the Priority Setting Partnerships used in research preparation stages such as those established in the United Kingdom by the James Lind Alliance [69]. The attempt to minimise this limitation was addressed by using broader search terms that may be all-encompassing of this research practice and by narrowing the selection by using robust and systematic removal of irrelevant records that did not support the research question exploration; this included limitations in framing the patient engagement outcomes by using the existing SPOR framework that broadly encompasses improved patient experience with the health system and health outcomes.

With respect to rigor in article selection, this study could have been further supported with an additional researcher to independently scan, identify, select and remove records from the yield to maximise integrity of the search process. The attempt to minimise this limitation was addressed by a clear and step-wise process to track articles included and excluded from the review as agreed upon by the Project Advisory Committee.

## Conclusion

The evidence resulting from this review suggests that engaging patients in health research does indeed have benefits as well as challenges. Factors that enable and limit the effectiveness of engagement across the research activity lifecycle were identified. While there is promising growth in the quantity and quality of research around engaging the patient across the research lifecycle, the findings from this review indicate that further mobilising interest in this promising practice by focusing research on developing and validating specific frameworks and tools is needed to better sustain patient engagement across the lifecycle of research with more rigour and stronger evidence about impact and outcomes. It is suggested that, by further increasing evidence that supports this practice, a paradigm shift is more likely to occur, normalising the patient’s role in research beyond that of ‘subject’ to that of a partner in improving patient health outcomes and addressing healthcare reform. Based on the findings from this scoping review, the Alberta SPOR SUPPORT Unit, Patient Engagement Platform, has identified three key recommendations:

1. Clarify the terminology of patient engagement in health research to illuminate expectations and understanding for patients, researchers, clinicians and policy-makers. The lack of consistency in terminology use and definitions only further adds to the confusion and complexity surrounding patient engagement in research, while diluting the possibility of achieving meaningful and successful engagement from all stakeholders [15, 42, 51].
2. Implement a predefined, validated framework to support and evaluate patient engagement in research. While the investment of the Canadian federal government in more patient engagement in health research is of value, it is timely for attention and resources to also be directed to developing, validating and implementing a framework for patient engagement in health research [15]. The framework applied to underpin patient engagement activities should be established prior to engaging patients and before execution of any research activity. This framework needs to be validated as relevant, effective and feasible, and should inform the development of practical tools adaptable in the local context [10]. This may require a reassessment of existing SPOR funding opportunities and outcomes to ensure development of a co-designed framework that focuses on evidence to support implementation and patient focused outcomes. A cluster of robust Canadian grounded theory studies can result in an integrated co-developed patient engagement framework that would include the processes and context of patient engagement to inform the practice and evaluation of patient engagement going forward.
3. Support development of evaluation frameworks and tools, and collection of robust evaluation data to measure near, intermediate and long-term outcomes. The impact of patient engagement should be captured in a standardised and valid way, whether it be through qualitative, quantitative or mixed-methods approaches to capture the complexity of this form of engagement [15]. Any tools developed should be informed by the qualitative data already collected. Evaluation should be continuous throughout the engagement process [10, 15, 56, 65]. Patient-reported outcome measures provide a novel opportunity to apply to this practice, and present a strong opportunity to leverage patient engagement in research in a very meaningful way to patients [54, 62]; this also includes documenting the context and process of engagement as fundamental components of the evaluation (i.e. funding, policy, physical environment or attitudes of those involved, how they are involved, procedures to promote success).

## Abbreviations

PCORI: Patient-Centered Outcome Research Institute’s
SPOR: Strategy for Patient-Oriented Research
SUPPORT: Support for People and Patient Oriented Research and Trials Unit
