# Correction to: Patient engagement in Canada: a scoping review of the ‘how’ and ‘what’ of patient engagement in health research

**DOI:** 10.1186/s12961-018-0296-y

**Published:** 2018-03-14

**Authors:** Elizabeth Manafo, Lisa Petermann, Ping Mason-Lai, Virginia Vandall-Walker

**Affiliations:** Patient Engagement Platform, Alberta SPOR SUPPORT Unit, Faculty of Health Disciplines, Athabasca University, 1 University Drive, Athabasca, Alberta T9S 3A3 Canada

## Correction

It has been highlighted that the original manuscript [1] contains a typesetting error in the name of Virginia Vandall-Walker. This was incorrectly captured as Virgnia Vandall-Walker in the original manuscript which has since been updated.
